# “Don’t Know” Responses for Nicotine Vaping Product Features among Adult Vapers: Findings from the 2018 and 2020 ITC Four Country Smoking and Vaping Surveys

**DOI:** 10.3390/ijerph18157928

**Published:** 2021-07-27

**Authors:** Nicholas J. Felicione, K. Michael Cummings, Shannon Gravely, David Hammond, Ann McNeill, Ron Borland, Geoffrey T. Fong, Richard J. O’Connor

**Affiliations:** 1 Roswell Park Comprehensive Cancer Center, Department of Health Behavior, Buffalo, NY 14263, USA; 2 Department of Psychiatry & Behavioral Sciences, Medical University of South Carolina, Charleston, SC 29425, USA; cummingk@musc.edu; 3 Department of Psychology, University of Waterloo, Waterloo, ON N2L 3G1, Canada; shannon.gravely@uwaterloo.ca (S.G.); geoffrey.fong@uwaterloo.ca (G.T.F.); 4 School of Public Health & Health Systems, University of Waterloo, Waterloo, ON N2L 3G1, Canada; david.hammond@uwaterloo.ca; 5 Department of Addictions, King’s College London, London WC2R 2LS, UK; ann.mcneill@kcl.ac.uk; 6 Shaping Public Health Policies to Reduce Inequalities and Harm (SPECTRUM), Nottingham NG5 1PB, UK; 7 School of Psychological Sciences, University of Melbourne, Melbourne, VIC 3010, Australia; rborland@unimelb.edu.au; 8 Cancer Council Victoria, Melbourne, VIC 3004, Australia; 9 Ontario Institute for Cancer Research, Toronto, ON M5G 0A3, Canada

**Keywords:** nicotine vaping products, electronic cigarette, knowledge, awareness, perceptions

## Abstract

Nicotine vaping products (NVPs) have evolved rapidly, and some vapers have difficulty reporting about their NVP. NVP knowledge may be important for providing accurate survey data, understanding the potential risks of NVP use, and assessing legal and regulated products. This paper examines current vapers who responded “don’t know” (DK) regarding their NVP features. Data are from adult daily/weekly vapers in Waves Two (2018, *n* = 4192) and Three (2020, *n* = 3894) of the ITC Four Country Smoking and Vaping Survey. Analyses assessed DK responses for NVP features (e.g., type/appearance, nicotine) and consumption. A DK index score was computed based on the percent of all features with DK responses, which was tested for associations with demographics, smoking/vaping status, NVP features, purchase location, and knowledge of NVP relative risks. NVP description and appearance were easily identified, but DK was more common for features such as nicotine content (7.3–9.2%) and tank/cartridge volume capacity (26.6–30.0%). DK responses often differed by vaping/smoking status, NVP type/appearance, purchase location, and country. Vapers who are younger, use box-shaped NVPs, purchase online, and exclusive daily vapers were associated with lower DK index scores. Higher DK index scores were associated with poorer knowledge of relative health risks of NVP use. The diversity of the NVP market and wide variation in how products are used makes it challenging to capture information from users about device features, such as nicotine content and capacity, in population surveys.

## 1. Introduction

Nicotine vaping products (NVPs) have rapidly evolved since their introduction to the global market in 2007, though all are similar in that they contain a battery, heating element, and a liquid that can be aerosolized and inhaled [1]. Initial ‘cigalike’ models were closed systems (i.e., non-refillable) that looked like cigarettes and allowed for little user modifiability. Next came tank-style NVPs, which are open systems (i.e., refillable) that allow the user more control of liquid constituents, such as flavor and nicotine concentration [1]. Tanks evolved into ‘mods’, which permitted the user to customize their NVP further, such as adjusting voltage/wattage and designing atomizers [2]. More recently established were pod or ‘cartridge’ NVPs, such as JUUL, which are typically less modifiable but also introduced newer features, such as nicotine salts [3]. Thus, the features and terminology of NVPs have changed since their emergence, though little research has assessed vapers’ knowledge of NVPs and factors associated with knowledge (i.e., demographic and NVP characteristics associated with knowledge) as they have evolved.

A standardized terminology for NVPs has not been established among users or researchers, which may make it difficult for vapers to identify or describe their NVPs and features in surveys. Early focus groups indicated that cigalike users expressed limited knowledge and were unable to describe differences between NVPs though they were familiar with basic features such as the ability to refill or recharge [4]. Researchers have used formal terms such as Electronic Nicotine Delivery Systems [5] and NVPs [6], or classified products into generations [2,7]. By contrast, some NVP users may use less technical terms such as ‘e-cigarette’ and ‘vape’, and less commonly use details such as brand name [5], though identification via brand name (e.g., JUUL) has become more common recently [8]. A lack of consistent terminology in communicating between consumers and researchers may be contributing to misreporting of NVP use and knowledge.

Some vapers also may also truly lack knowledge regarding NVP features, which can impact the data quality provided in surveys. Studies have reported that some vapers do not know if NVPs contain nicotine [9], or if they do, their specific nicotine concentration [10,11,12,13], whether their nicotine concentration is consistent with regulatory limits (i.e., 20 mg/mL in England) [11,14], or incorrectly report nicotine concentration [15]. Vapers also may have trouble distinguishing between nicotine contents presented as mg/mL and percent [16]. Up to half of vapers do not know about the chemical constituents or ingredients of NVPs [9,10,17], with some believing it only contains harmless water vapor [17]. Additionally, over half of adult vapers in one survey were unable to report device voltage or atomizer resistance or reported nonsensical values [12], and 43.5% of young adult pod-style vapers were unaware if they use the appropriate brand of cartridges for their NVP [15]. Despite using NVPs, many vapers appear to lack in-depth knowledge about their NVP features and constituents.

High rates of “don’t know” (DK) responses in surveys asking about NVP features is problematic as it raises questions about the accuracy of data used for tracking consumer product use. Survey makers must use appropriate terminology and response options to maximize validity of consumer responses; otherwise misunderstanding may lead to inaccurate estimates of popular NVPs and how they are used. Second, NVP knowledge is relevant to health for understanding exposure to addictive and harmful constituents. This relationship has been observed for cigarette filter ventilation, in which smokers who are aware of filter ventilation were more likely than those unaware to be concerned about lung cancer [18]. Regarding NVPs, nicotine concentration and power are associated with nicotine and toxicant delivery [19] and nicotine dependence [20], so unawareness of these features may be associated with unawareness of addiction risk and potential harms of NVPs. Finally, NVP knowledge is important for identifying legal and regulated products, such as following the European Union Tobacco Products Directive that limits nicotine content (20 mg/mL, ~10% DK) and NVP capacity (2 mL, ~40% DK) [11]. Similarly, lacking knowledge of NVP features may suggest to regulators that better labelling and communication strategies are needed to ensure vapers are informed consumers.

The overall aim of this paper is to assess who chooses DK to questions about NVP features (with response options), and whether DK for NVP features has implications for knowledge of addiction and relative health risks of NVP use. This aim is explored using a repeat cross-sectional analysis of a sample of current vapers in 2018 and 2020. We examined whether DK responses about NVPs differs based on vaping/smoking status, device type, purchase location, and country, and examined user and device characteristics associated with DK for NVP features. Finally, we tested if DK for NVP features was associated with DK regarding vaping risks. We specifically focused on those who chose DK (valid response) and did not aim to verify accuracy of other responses.

## 2. Materials and Methods

### 2.1. ITC Overview

The ITC Four Country Smoking and Vaping Survey (ITC 4CV) is a longitudinal cohort study that consists of four parallel online surveys conducted in Canada, the United States, England, and Australia [21]. In addition to respondents retained from the ITC Four Country Survey (predecessor of ITC 4CV) [22], adults (≥18 years) were recruited by commercial panel firms in each country beginning at Wave 1 (July–November 2016) as cigarette smokers (≥100 lifetime), recent ex-smokers (quit within ≤2 years), and at-least-weekly NVP users (ITC Project, 2020). For the current study at Wave 2 (2018), Wave 1 respondents were invited back, and those lost to attrition were replenished.

### 2.2. Sample

Data for this current study come from 4192 (Wave 2: February to July 2018; 34.4% recontact, 65.6% newly recruited) and 3894 (Wave 3: February to June 2020; 45.7% recontact, 54.3% newly recruited) vapers who reported current daily (66.0% 2018; 69.8% 2020) or weekly (34.0% 2018; 30.2% 2020) NVP use.

### 2.3. Measures

Questions referred to the vapers most commonly used “e-cigarette/vaping device.” All responses were dichotomized into valid (any valid response) and “don’t know” (DK). Vapers who refused to answer were considered missing.

#### 2.3.1. Vaping/Smoking Status

NVP and cigarette use were dichotomized as daily or nondaily (i.e., weekly). These variables were combined to create six use status categories: (1) exclusive daily vaper, (2) exclusive nondaily vaper, (3) dual daily user (daily NVP/daily cigarette), (4) predominant vaper (daily NVP/nondaily cigarette), (5) predominant smoker (nondaily NVP/daily cigarette), and (6) concurrent nondaily user (nondaily NVP/nondaily cigarette) [23].

#### 2.3.2. Purchase Location

All vapers were asked about where they purchased their last NVP or e-liquid. Responses were categorized into Online, Vape Shop, or Other Retail [24].

#### 2.3.3. NVP Features

Supplemental Table S1 provides the questions and response options for each NVP feature.

1. NVP description: Vapers were asked to describe their NVP as being disposable, using pre-filled cartridges/pods, using a refillable tank, or DK.
2. NVP appearance: In 2018, vapers were asked to describe the appearance of their NVP as cigarette-like, cigarette-like but different color, pen-like, box-like, other, or DK. In 2020, an additional option for USB/flash drive-like was added.
3. NVP Brand: Vapers were asked to report on their NVP brand by typing the brand and then choosing from a list of brand names, selecting other and providing a brand, or DK. In 2020, tank users were not provided with a brand list and were asked to type in their brand. A researcher reviewed the reported tank brands and brands coded as “other” and coded as DK if the response was not an NVP brand (e.g., Chantix, iQOS, random letters).
4. Adjustable voltage: Cartridge and tank users were asked if their power/voltage was adjustable and could select yes, no, or DK.
5. NVP capacity: Cartridge and tank users were asked to report on the volume/capacity of their tank/cartridge in mL, with response options providing size ranges (e.g., <1 mL, 1–1.5 mL, etc.) or DK.
6. Nicotine content: In 2018, vapers were asked to report on their nicotine content with response options including both mg/mL and percent (e.g., “0 mg/mL (0%)”). In 2020, vapers were first asked if they prefer to report in mg/mL or percent and then all response options corresponded with their preferred unit of measurement. In 2020 only, vapers were asked if they have ever used nicotine salts and could reply “yes”, “no”, or “don’t know”.

#### 2.3.4. NVP Consumption

Consumption, a measure of NVP use, was assessed differently for disposable/cartridge and tank users. Disposable and cartridge users were asked to report on how many NVPs they use each week, and tank users were asked to report on how long their most recently purchased e-liquid bottle will last.

#### 2.3.5. DK Index

DK responses for each NVP variable were used to create a DK index, which is the percent of items with a DK response. A maximum value of 100 indicates a DK response for every item, while a minimum value of 0 indicates all valid responses. The denominator for the DK index (i.e., number of items) differed based on wave and response patterns. There were 7 relevant items in 2018 (NVP description, NVP appearance, nicotine content, adjustable voltage, capacity, brand, consumption) and 8 relevant items in 2020 (added nicotine salts). Disposable NVP users were not asked adjustable voltage or capacity, reducing the number of relevant items asked to 5 in 2018 and 6 in 2020. DK index scores were not significantly correlated with the number of variables for which a vaper had missing data (r = −0.002, *p* = 0.89), suggesting that scores were not impacted by missingness. DK index scores ranged from 0–71.43 (M = 9.19, SD = 12.53) in 2018 and 0–75.00 (M = 7.75, SD = 10.99) in 2020. DK index scores were positively skewed and were log-transformed after adding 1 to each because the data included zeroes. Log-transformed DK index scores were used for statistical tests, though untransformed means are reported for ANOVAs to ease interpretation.

#### 2.3.6. NVP Relative Risks

Vapers were asked three questions about the relative risks of vaping compared to regular cigarettes: harmfulness, second-hand smoke/vapor harmfulness, and addictiveness. Each question had the responses: (1) much less, (2) somewhat less, (3) equally, (4) somewhat more, (5) much more, (6) refused, (7) DK. Relative harmfulness and secondhand harmfulness also were used to assess correct perceptions. Responses of “much less” or “somewhat less” were defined as correct and all other responses were defined as incorrect [25,26].

### 2.4. Data Analyses

Cross-sectional analyses were weighted to the country-representative samples using the rescaled cross-sectional weight for current NVP users at each wave. For all analyses, vapers who reported DK were classified as DK, any valid response was classified as knowledge, and refused was classified as missing.

Chi-squared analyses were used to test if DK percentages differed by vaping/smoking status, NVP description, NVP appearance, purchase location, and country (*p* < 0.05). One-way ANOVAs were used to test if DK index scores differed by these same variables (*p* < 0.05), with Tukey’s HSD used for post-hoc comparisons (raw M ± SE presented). Generalized linear models were used to predict DK index scores based on demographic factors (country, age, education, income, sex, race/ethnicity), vaping/smoking status, NVP description, NVP appearance, purchase location, and controlled for recontact vs. replenishment sampling. All predictors were entered as categorical variables. Separate models were used to predict DK index scores in 2018 and 2020. Six binary logistic regressions were used to test if DK index scores predicted a DK response (valid as referent) to relative risk perceptions (addictive, harmful, secondhand harm) in 2018 and 2020 when controlling for the variables included in the previous models. Finally, four binary logistic regressions were used to test if DK index scores predicted a correct response to relative harm and secondhand harm perceptions in 2018 and 2020 when controlling for the same variables. We also used chi-squared analyses to test whether those who know versus DK nicotine differed in DK responses about NVP addiction risk. All analyses were conducted using IBM SPSS Statistics version 25 (IBM, Armonk, NY, USA).

## 3. Results

Table 1 provides the overall DK percentages for each NVP feature. Fewer than 0.4% of vapers DK their NVP description or appearance in 2018 and 2020. Due to low rates of DK, NVP description and appearance are not highlighted throughout the results. Brand was less easily identified, with DK reported by 13.6% in 2018 and 9.1% in 2020. NVP capacity was the least known feature, with 30% DK in 2018 and 26.6% in 2020. All other features (adjustable voltage, nicotine content, nicotine salts, consumption) had less than 10% DK, with the lowest DK for adjustable voltage. DK index scores (i.e., % of features that a vaper responded DK) were also below 10 in both years. DK reduced by several percentage points from 2018 to 2020 for all features except NVP appearance and adjustable voltage.

### 3.1. DK by Vaping/Smoking Status

Table 1 summarizes the results for each item by vaping/smoking status. DK responses for brand differed by vaping/smoking status in 2018 and 2020 (*p* < 0.001), being lower for daily users (particularly exclusive daily vapers) than nondaily users. A similar result was observed for adjustable voltage, in which DK was lowest in exclusive daily vapers and predominant vapers.

DK for capacity differed by vaping/smoking status in 2018 (*p* < 0.05), but not 2020 (*p* = 0.07). DK percentages were high for capacity, even among exclusive daily vapers (26.9–31.9%).

DK responses differed by vaping/smoking status for nicotine content in 2018 and 2020 (*p* < 0.001), again with lower DK percentages among daily vapers compared to nondaily. Exclusive daily vapers also had the lowest DK percentage for nicotine salts in 2020. DK for consumption also differed by vaping/smoking status in 2018 and 2020 (*p* < 0.001). In 2018, exclusive (daily and nondaily) and predominant vapers had lower DK than other dual use groups, though DK increased for exclusive nondaily vapers and concurrent nondaily users in 2020.

DK index scores differed by vaping/smoking status in 2018 (*F* (54,185) = 4.00, *p* = 0.001) and 2020 (*F* (53,887) = 20.27, *p* < 0.001). In 2018, exclusive daily vapers had a lower DK index score than predominant smokers (*p* = 0.002) and concurrent nondaily users (*p* < 0.05). In 2020, exclusive daily vapers had a lower DK index score than all vaping/smoking statuses (*p* < 0.001) except predominant smokers. Additionally, predominant vapers had a lower score than exclusive nondaily vapers (*p* < 0.05).

### 3.2. DK by NVP Features

#### 3.2.1. NVP Description

Table 2 summarizes the results for each item by NVP description. DK responses for brand differed by NVP description in 2018 (*p* < 0.001) and 2020 (*p* = 0.002), though the rank order changed. In 2018, the lowest DK percentages were observed for tank users (12.4%), though it was lowest among cartridge users (6.9%) in 2020.

DK percentages for adjustable voltage differed by NVP description in 2018 and 2020 (*p* < 0.001), with higher percentages among cartridge users (4.9% 2018, 7.9%; 2020) than tank users (1.2% 2018, 2.8%; 2020). A similar pattern was observed for capacity.

DK responses for nicotine content also differed by NVP description in 2018 and 2020 (*p* < 0.001). In both waves, tank users had the lowest DK percentage, though a large reduction in DK was observed for cartridge users from 2018 to 2020. Tank users also reported lower DK percentages for nicotine salts in 2020. DK for consumption differed by NVP description in 2020 (*p* < 0.001), but not 2018. Generally, disposable users reported the highest DK in both years.

DK index scores differed by NVP description in 2018 (*F* (24,154) = 37.24, *p* < 0.001) and 2020 (*F* (23,883) = 17.17, *p* < 0.001). Cartridge users had higher DK index scores than tank and disposable users in 2018 (*p* < 0.001) and 2020 (*p* < 0.001 and <0.05, respectively).

#### 3.2.2. NVP Appearance

Table 3 summarizes the results for each item by NVP appearance. DK percentages for all features differed by NVP appearance in 2018 and 2020 (*p* < 0.001), except consumption in 2018. DK for NVP description was high (5.2%) among vapers who reported “other” for NVP appearance. In both years, the highest DK percentages for brand were reported by cigalike and cigalike-different color users.

DK for adjustable voltage was generally highest for both cigalikes and lowest for box-shaped in both years, with capacity following a similar trend.

For nicotine content in 2018 and 2020, box-shaped and pen-shaped users generally responded DK less than users of cigalikes and ”other”. DK percentages for nicotine salts differed by NVP appearance (*p* < 0.001), with the highest reporting among cigalike-different color (8.7%), and cigalike/USB-shaped (7.3%), while lowest for box-shaped (3.1%). For consumption in 2020, DK percentages were highest among cigalike groups (9.3–14.0%).

DK index scores differed by NVP appearance in 2018 (*F* (44,181) = 18.81, *p* < 0.001) and 2020 (*F* (53,875) = 8.79, *p* < 0.001). Box-shaped users had a lower DK index score than all other categories in 2018 (*p* < 0.001) and 2020 (*p* < 0.05).

### 3.3. DK by Purchase Location

Table 4 summarizes the results for each item by purchase location. DK for brand differed by purchase location in 2018, with the lowest DK reported for online stores (7.7%), followed by vape shops (14.2%) and other retail (17.2%), though these differences were not sustained in 2020.

DK for NVP capacity and nicotine content differed by purchase location in 2018 and 2020 in the same direction as brand (i.e., lowest online, highest other retail). Some results changed between waves, such as vape shops having the lowest DK for adjustable voltage in 2018 (1.7%) but the highest in 2020 (5.5%). A similar change was observed with consumption for other retail, having the highest DK percentage in 2018 (11.8%) but lowest in 2020 (3.5%).

DK index scores differed by purchase location in 2018 (*F* (24,160) = 33.65, *p* < 0.001) and 2020 (*F* (23,524) = 12.78, *p* < 0.001). In 2018 and 2020, other retail purchasers had a higher DK index score than online or vape shop (*p* < 0.001). Additionally in 2018, online purchasers had a lower DK index score than vape shop (*p* < 0.05).

### 3.4. DK by Country

Supplemental Table S2 summarizes the results for each item by country. Large differences in DK were observed for brand in 2018 (6.6% in AU, 23.4% in CA), though differences were less distinct in 2020 (6.9–11.1%). In 2018 and 2020, AU had relatively lower levels of DK for capacity (11.6–14.9%) compared to other countries (26.1–34.3%). Regarding nicotine content, reductions in DK from 2018 to 2020 were observed in CA, increases observed in AU, and stability in US and EN. Reductions in DK for consumption from 2018 to 2020 were observed for all countries except AU, which increased in DK percentage.

DK index scores differed by country in 2018 (*F* (34,188) = 17.46, *p* < 0.001) and 2020 (*F* (33,889) = 5.32, *p* < 0.01). In 2018, DK index scores were lower in AU than all other countries (<0.001) and were higher in CA than EN (*p* < 0.01) and US (*p* < 0.05). In 2020, DK index scores were lower in CA than US (*p* < 0.01) and EN (*p* < 0.05).

### 3.5. Factors Associated with DK Index

Table 5 displays regression models to predict DK index scores in 2018 and 2020. In 2018, lower DK index scores were associated with residing in AU (ref = US), being 18–24 and 25–39 years old (ref = 55+), using a box-shaped or pen-shaped NVP (ref = cigalike), and purchasing online (ref = other retail). Higher DK index scores were associated with low and moderate education (ref = high), being female, and using cartridges or tanks (ref = disposable).

In 2020, lower DK index scores were associated with residing in AU and CA (ref = US), any age group below 55+, using box-shaped NVPs (ref = cigalike), and purchasing online or at vape shops (ref = other retail). Higher DK index scores were associated being female, any vaping/tobacco status except predominant vapers (ref = exclusive daily vapers), and tank or cartridge NVPs (ref = disposable).

### 3.6. DK and Accuracy for NVP Relative Risks

Increased DK index scores were associated with increased odds of reporting DK for all three NVP relative risks (i.e., compared to cigarettes) in both years: addictiveness in 2018 (OR = 2.96, 95% CI = 2.26–3.87) and 2020 (OR = 1.89, 95% CI = 1.45–2.45); harmfulness in 2018 (OR = 2.19, 95% CI = 1.74–2.77) and 2020 (OR = 2.55, 95% CI = 1.87–3.48); secondhand harmfulness in 2018 (OR = 2.33, 95% CI = 1.80–3.01), and 2020 (OR = 1.72, 95% CI = 1.38–2.16). Additionally, increased DK index scores were associated with reduced odds of reporting a correct answer (i.e., much or somewhat less harmful) in 2020 regarding NVP relative harmfulness (OR = 0.72, 95% CI = 0.62–0.84) and secondhand harmfulness (OR = 0.81, 95% CI = 0.70–0.93). However, DK index scores were not significantly associated with odds of reporting a correct answer in 2018 regarding NVP relative harmfulness (OR = 0.88, 95% CI = 0.77–1.00) and secondhand harmfulness (OR = 0.91, 95% CI = 0.80–1.03).

Additionally, DK for nicotine content was associated with DK for NVP relative addictiveness in 2018 (*Χ*^2^ (1) = 23.52, *p* < 0.001) and 2020 (*Χ*^2^ (1) = 35.52, *p* < 0.001). In 2018, DK for NVP relative addictiveness was reported by 10.4% of those who DK and 4.6% of those who knew nicotine content. In 2020, DK for NVP relative addictiveness was reported by 13.8% of those who DK and 5.1% of those who knew nicotine content.

## 4. Discussion

Nearly all current vapers were able to identify basic NVP features, such as appearance and how e-liquids used was stored (i.e., disposable/not-refillable, pre-filled cartridges/pods, refillable tanks). Many current vapers reported DK when asked about brand used, although DK responses decreased from 2018 to 2020, suggesting that NVP brands have become more salient. Reporting of device capacity had the highest levels of DK of all NVP features. Capacity may be useful for vapers to monitor their consumption but may be less important for vapers to understand their potential nicotine and toxicant exposure. Regarding features that may impact nicotine/toxicant delivery (i.e., nicotine content, salt, adjustable voltage, consumption), nicotine content and consumption had relatively higher DK compared to adjustable voltage and nicotine salts, though all were below 10% DK. DK responses to questions about NVP features generally differed by vaping and smoking status, NVP type, purchase location, and country. DK responses decreased from 2018 to 2020 for most NVP features, perhaps indicating increased awareness of product features and reflecting a shift in the types of NVPs consumed with an increase in disposables and refillable cartridge/pods systems among current vapers in the US and Canada [3]. Importantly, current vapers who were able to report more about their product features were better informed about the relative risks of NVPs compared to cigarettes.

Nicotine content is a target of regulations (e.g., Tobacco Products Directive limits maximum nicotine concentration at 20mg in EN; no nicotine in AU) [27,28], and also has implications for abuse liability [29,30], dependence [20,31], and smoking cessation [32]. DK responding for nicotine content was consistent with previous surveys of adult vapers [11,12,14], and seem broadly consistent with country regulations. For instance, the US, which has the least intensive enforcement of regulations, had relatively high DK percentages in 2018 and 2020. In contrast, the Tobacco Products Directive includes restrictions on nicotine content (≤20 mg/mL) [27], therefore may promote better awareness of this feature as observed in EN. In CA, the Tobacco and Vaping Products Act introduced labelling requirements during 2018 data collection [33], which may explain the reduction in DK for nicotine content in CA from 2018 to 2020. DK for nicotine concentration was reported less frequently for tank users compared to cartridge and disposable users. Tanks are open systems that require vapers to purchase refill nicotine solutions separately, which may lead to greater attention to product details and enhanced user knowledge compared to closed systems. For integrated systems, the focus may be more on the device or brand, e.g., some vapers may be attracted to popular brand names such as JUUL. These results suggest that labelling and packaging of products can be improved to communicate product features, particularly for NVPs pre-filled with e-liquid. Better labelling may also be necessary to increase awareness of capacity, as almost 30% of vapers in EN reported DK for capacity despite regulatory limits of ≤2 mL [11].

Generally, daily vapers reported less DK of NVP features compared to nondaily users, with the least DK observed among exclusive daily vapers. These findings are in contrast with other data suggesting that those who vape more frequently are more likely to lack knowledge of the contents of NVPs [34]. However, between-study differences may be a result of survey items and the depth of knowledge solicited. For instance, the ITC 4CV study asks about nicotine concentration with provided response options, while other surveys may include questions that require more depth of knowledge (e.g., “I can list all the ingredients in a vape pod”) [34], or focus on perceptions rather than objective NVP features. Survey makers should consider the granularity of detail needed when designing questions, as question wording and specificity of response options may influence DK responses.

Several demographic and NVP use characteristics were associated with DK index scores. In 2018, AU was associated with lower DK index scores compared to US, along with CA in 2020. As suggested for nicotine content, the lack of comprehensive regulations in US (e.g., nicotine contents, tank capacities, labelling requirements) may lead to uncertainty in US vapers about their NVP features. Younger ages and using box-shaped NVPs were consistently associated with lower DK index scores. Thus, these open system NVPs may lead users to pay more attention to and have enhanced knowledge of features. Those who purchase their NVPs online also had consistently lower DK index scores than those who purchase at other retail, with vape shops falling in the middle. Importantly, those with higher DK index scores were more likely to respond DK or incorrectly about relative risks of NVPs compared to cigarettes, demonstrating a concerning implication for lacking awareness about NVP features. Accurate NVP harm perceptions are associated with NVP use among smokers, so this knowledge may be important for facilitating product switching [35].

Several limitations of the current study highlight the need for continuing research. First, knowledge was characterized by a valid response versus DK, but was not assessed for accuracy (e.g., did NVP type match brand). Validation of responses is difficult, as it cannot be done consistently across all brands (e.g., JUUL sells one device type; Blu sells three device types; Smok sells many device types) and may require knowledge of vaper behaviors that were not assessed (e.g., modifying products, circumventing regulations). Second, the reasons for DK responses are unknown. Vapers may use different terminology than researchers, and DK may have been due to a lack of understanding the questions or response options, or conceptualizing the information in different ways (e.g., nicotine strengths as low, medium, high; tank capacity as large or small). Continued research assessing vaper terminology is important to ensure that researchers are using similar language to maximize validity of survey responses. Vapers who use multiple NVP devices and/or liquids and may respond DK when forced to choose one option, although we asked for their most commonly used NVP. Additionally, some questions may not capture the depth or specificity of user knowledge. For example, vapers were asked if their NVP had adjustable voltage (global assessment), but not their exact power settings (more specific assessment) which may be unknown by a large percentage of vapers [12]. Finally, changes in survey wording, response options, or interviewing strategies may explain some differences in results between 2018 and 2020. For example, vapers may have trouble understanding nicotine strengths in mg and % [16]. Nicotine content questions were separated by mg/mL or % in 2020 (vs. combined in 2018), and 2020 generally had lower DK percentages than 2018. Thus, this change may have led to less confusion in responding.

## 5. Conclusions

Overall, current vapers were able to report basic NVP features, though brand was less well known. However, consumers were more challenged in reporting specific features of their NVP such as nicotine content and capacity. Not surprisingly, daily vapers and those reporting purchasing from online and vape shops were less likely to report DK responses. Since NVP features are known to impact nicotine delivery and may impact health risks, population surveys need to develop and validate better ways to assess the NVPs consumers are using. Finally, it may be important to ensure vapers are aware of their NVP features, as those who were better informed were also more aware of the relative risks of NVPs compared to cigarettes.

## Figures and Tables

**Table 1:** DK percentages overall and by vaping/smoking status.

	Exclusive Daily Vaper	Exclusive Nondaily Vaper	Predominant Vaper	Predominant Smoker	Dual Daily User	Concurrent Nondaily User	Overall		
*n* at 2018	1640	339	496	658	747	311	4192		
*n* at 2020	1896	474	217	482	606	219	3894	*X* ^2^ *or F*	*p*
NVP Description									
2018 (*n* = 4172)	0.2	1.2	0.0	0.2	0.5	0.6	0.3	10.87	0.054
2020 (*n* = 3890)	0.0	0.2	0.0	0.2	0.0	0.5	0.1	8.35	0.14
NVP Appearance									
2018 (*n* = 4190)	0.0	0.0	0.2	0.0	0.3	0.0	0.0	7.30	0.20
2020 (*n* = 3893)	0.1	0.6	0.0	0.2	0.7	0.5	0.3	8.20	0.15
Brand									
2018 (*n* = 3809)	7.7	23.0	9.9	18.7	15.2	27.7	13.6	134.60	<0.001
2020 (*n* = 3858)	4.5	14.1	7.4	14.2	12.6	19.9	9.1	114.70	<0.001
Adjustable Voltage									
2018 (*n* = 3867)	0.6	2.5	1.9	4.4	3.7	3.1	2.2	41.27	<0.001
2020 (*n* = 3817)	2.2	9.3	2.0	7.7	6.7	8.9	4.7	69.92	<0.001
Capacity									
2018 (*n* = 3860)	31.9	25.5	32.5	30.1	26.0	29.2	30.0	12.31	0.031
2020 (*n* = 3563)	26.9	31.3	26.2	26.5	24.0	20.4	26.6	10.17	0.07
Nicotine Content									
2018 (*n* = 4188)	6.4	13.9	7.5	12.9	9.4	13.5	9.2	43.71	<0.001
2020 (*n* = 3855)	3.1	8.9	8.3	15.4	9.0	16.5	7.3	126.00	<0.001
Nicotine Salt									
2018 (n/a)	n/a	n/a	n/a	n/a	n/a	n/a	n/a	n/a	n/a
2020 (*n* = 3888)	4.3	5.7	5.1	5.8	9.3	6.8	5.6	22.23	<0.001
Consumption									
2018 (*n* = 2768)	7.2	5.1	5.9	12.7	16.5	10.8	9.7	49.74	<0.001
2020 (*n* = 2297)	2.0	11.0	6.7	12.2	11.7	16.5	6.4	98.57	<0.001
DK Index									
2018 (*n* = 4191)	7.74 (0.26)	10.41 (0.80)	8.48 (0.53)	10.83 (0.53)	9.83 (0.49)	11.63 (0.85)	9.19 (0.19)	4.00	0.001
2020 (*n* = 3894)	5.51 (0.20)	9.68 (0.55)	6.80 (0.65)	10.05 (0.59)	9.02 (0.50)	10.95 (0.98)	7.75 (0.18)	20.27	<0.001

**Table 2:** DK percentages by NVP description.

	Disposable	Pre-Filled Cartridge	Refillable Tank		
*n* in 2018	288	992	2878		
*n* in 2020	315	1323	2249	*X* ^2^ *or F*	*p*
NVP Appearance					
2018	0.7	0.0	0.0	16.73	<0.001
2020	0.0	0.2	0.3	1.72	0.42
Brand					
2018	17.1	17.0	12.4	14.58	<0.001
2020	12.2	6.9	10.0	12.87	0.002
Adjustable Voltage					
2018	n/a	4.9	1.2	48.33	<0.001
2020	n/a	7.9	2.8	49.35	<0.001
Capacity					
2018	n/a	36.1	27.9	23.30	<0.001
2020	n/a	28.2	25.6	2.85	0.091
Nicotine Content					
2018	15.4	19.6	4.9	204.73	<0.001
2020	13.1	9.5	5.1	40.88	<0.001
Nicotine Salt					
2018	n/a	n/a	n/a	n/a	n/a
2020	5.7	8.0	4.2	22.71	<0.001
Consumption					
2018	12.6	8.9	9.7	3.58	0.17
2020	11.1	7.7	4.4	17.40	<0.001
DK Index					
2018	9.08 (0.76)	12.28 (0.45)	8.01 (0.21)	37.24	<0.001
2020	6.83 (0.64)	8.69 (0.32)	6.81 (0.22)	17.17	<0.001

**Table 3:** DK percentages by NVP appearance.

	Cigalike	Cigalike, Diff Color	USB/ FlashDrive	Pen-Shaped	Box-Shaped	Other		
*n* in 2018	484	369	n/a	1436	1782	116		
*n* in 2020	275	267	1008	818	1387	126	*X* ^2^ *or F*	*p*
NVP Description								
2018	0.2	0.0	n/a	0.4	0.1	5.2	85.97	<0.001
2020	0.4	0.0	0.0	0.0	0.0	0.0	13.21	0.022
Brand								
2018	22.0	18.0	n/a	14.4	10.6	7.8	47.03	<0.001
2020	17.0	12.2	5.4	9.7	9.5	7.2	40.39	<0.001
Adjustable Voltage								
2018	5.2	4.1	n/a	2.5	0.9	1.9	34.97	<0.001
2020	7.0	7.6	6.4	4.7	2.2	9.6	37.85	<0.001
Capacity								
2018	45.8	31.7	n/a	31.2	25.5	37.7	60.46	<0.001
2020	28.1	13.9	33.0	29.4	20.9	40.0	73.12	<0.001
Nicotine Content								
2018	11.2	18.7	n/a	9.8	5.5	21.6	92.81	<0.001
2020	12.8	13.3	7.0	6.2	5.4	12.6	40.70	<0.001
Nicotine Salt								
2018	n/a	n/a	n/a	n/a	n/a	n/a	n/a	n/a
2020	7.3	8.7	7.3	6.5	3.1	4.8	29.72	<0.001
Consumption								
2018	10.8	8.5	n/a	11.7	7.8	7.7	9.46	0.051
2020	9.3	14.0	5.2	5.4	5.8	5.1	21.44	0.001
DK Index								
2018	11.95 (0.65)	11.06 (0.73)	n/a	9.91 (0.33)	7.19 (0.26)	12.69 (1.29)	18.81	<0.001
2020	9.33 (0.76)	8.41 (0.71)	7.88 (0.33)	7.94 (0.39)	5.92 (0.26)	9.89 (1.21)	8.79	<0.001

**Table 4:** DK percentages by purchase location.

	Online	Vape Shop	Other Retail		
*n* in 2018	1048	1419	1697		
*n* in 2020	1027	1401	1099	*X* ^2^ *or F*	*p*
NVP Description					
2018	0.0	0.0	0.8	20.67	<0.001
2020	0.0	0.0	0.0	n/a	n/a
NVP Appearance					
2018	0.0	0.0	0.2	4.36	0.11
2020	0.2	0.2	0.2	0.03	0.98
Brand					
2018	7.7	14.2	17.2	45.72	<0.001
2020	7.0	8.1	9.9	5.85	0.054
Adjustable Voltage					
2018	1.9	1.7	2.7	3.69	0.16
2020	3.5	5.5	2.9	10.74	0.005
Capacity					
2018	24.6	27.1	36.1	44.97	<0.001
2020	21.7	25.1	34.3	42.75	<0.001
Nicotine Content					
2018	5.5	7.7	12.5	43.52	<0.001
2020	4.2	6.7	8.2	14.29	0.001
Nicotine Salt					
2018	n/a	n/a	n/a		
2020	4.7	3.6	7.3	17.41	<0.001
Consumption					
2018	8.1	8.0	11.8	8.16	0.017
2020	6.0	6.5	3.5	7.98	0.018
DK Index					
2018	6.83 (0.32)	8.40 (0.32)	11.19 (0.34)	32.65	<0.001
2020	5.89 (0.28)	6.94 (0.28)	8.21 (0.33)	12.78	<0.001

**Table 5:** Regression models to predict DK index scores in 2018 and 2020. Bolded values indicate significant predictors (*p* < 0.05).

2018				2020			
	B	SE	*p*		B	SE	*p*
Country				Country			
AU	−0.20	0.05	<0.001	AU	−0.11	0.05	0.032
CA	0.05	0.03	0.14	CA	−0.11	0.04	0.003
EN	−0.004	0.03	0.89	EN	−0.04	0.03	0.20
US	REF			US	REF		
Age				Age			
18–24	−0.10	0.04	0.013	18–24	−0.13	0.04	0.001
25–39	−0.12	0.03	<0.001	25–39	−0.13	0.03	<0.001
40–54	−0.04	0.03	0.21	40–54	−0.09	0.03	0.002
55+	REF			55+	REF		
Education				Education			
Low	0.13	0.04	<0.001	Low	0.02	0.04	0.63
Moderate	0.11	0.03	<0.001	Moderate	−0.02	0.03	0.54
High	REF			High	REF		
Income				Income			
Low	0.02	0.03	0.51	Low	−0.04	0.03	0.11
Moderate	0.02	0.03	0.37	Moderate	−0.04	0.03	0.10
High	REF			High	REF		
Sex				Sex			
Female	0.07	0.02	0.002	Female	0.14	0.02	<0.001
Male	REF			Male	REF		
Ethnicity				Ethnicity			
White	0.04	0.03	0.27	White	0.05	0.03	0.09
Nonwhite	REF			Nonwhite	REF		
NVP Status				NVP Status			
Concurrent Nondaily Users	0.10	0.05	0.055	Concurrent Nondaily Users	0.23	0.06	<0.001
Dual Daily Users	−0.02	0.03	0.54	Dual Daily Users	0.16	0.03	<0.001
Predominant Smokers	−0.01	0.03	0.81	Predominant Smokers	0.18	0.04	<0.001
Predominant Vapers	0.06	0.04	0.10	Predominant Vapers	0.05	0.05	0.37
Exclusive Nondaily Vapers	−0.05	0.04	0.21	Exclusive Nondaily Vapers	0.20	0.04	<0.001
Exclusive Daily Vapers	REF			Exclusive Daily Vapers	REF		
NVP Type				NVP Type			
Refillable Tank	0.14	0.05	0.01	Refillable Tank	0.27	0.05	<0.001
Pre-filled Cartridge	0.24	0.05	<0.001	Pre-filled Cartridge	0.23	0.05	<0.001
Disposable	REF			Disposable	REF		
NVP Appearance				NVP Appearance			
All Others	−0.10	0.08	0.18	All Others	−0.01	0.08	0.90
Box-shaped	−0.20	0.05	<0.001	Box-shaped	−0.15	0.05	0.007
Pen-shaped	−0.14	0.05	0.001	Pen-shaped	−0.07	0.06	0.18
USB/Flash Drive	n/a	n/a	n/a	USB/Flash Drive	−0.002	0.05	0.97
Cigalike, Diff Color	−0.10	0.05	0.055	Cigalike, Diff Color	−0.04	0.06	0.54
Cigalike	REF			Cigalike	REF		
Purchase Location				Purchase Location			
Online	−0.13	0.03	<0.001	Online	−0.11	0.03	<0.001
Vape Shop	−0.05	0.03	0.07	Vape Shop	−0.06	0.03	0.026
Other Retail	REF			Other Retail	REF		

## Data Availability

Data are available by request from itcproject.org/request-data-form/.

## Supplementary Materials

The following are available online at https://www.mdpi.com/article/10.3390/ijerph18157928/s1, Table S1: NVP questions and response options in ITC 4CV W2 (2018) and W3 (2020), Table S2: DK percentages by country.
