# Supplementary material for: “Don’t Know” Responses for Nicotine Vaping Product Features among Adult Vapers: Findings from the 2018 and 2020 ITC Four Country Smoking and Vaping Surveys
Source: Int J Environ Res Public Health. 2021 Jul 27;18(15):7928. doi: 10.3390/ijerph18157928 (PMC8345716; doi:10.3390/ijerph18157928)
Supplement: Supplementary file 1 [file ijerph-18-07928-s001.zip › ijerph-1250056-supplementary.pdf]

Supplemental Table S1. NVP questions and response options in ITC 4CV W2 (2018) and W3 (2020).

Variable	Wave	Question	Response Options
NVP Description	2018, 2020	Which of the following best describes the type…	1) Disposable, not-refillable (non-rechargeable)<br>2) Replaceable, pre-filled cartridges/pods (rechargeable)<br>3) Tank that you fill with liquids (rechargeable)<br>4) Refused<br>5) DK
NVP Appearance	2018	Which of the following best describes the appearance…	1) Looked like an ordinary cigarette, including shape, size, and colour<br>2) Looked similar in shape and size to an ordinary cigarette, but was a different colour<br>3) Looked similar in shape to a pen, but not necessarily round, and was pen-sized or larger<br>4) Looked like a box-shaped battery (that fit in the palm of my hand) with a mouthpiece<br>5) Looked different than any of the response options described above<br>6) Refused<br>7) DK
NVP Appearance	2020	Which of the following best describes the appearance…	Added option "Looked similar to a USB/flash drive"
NVP Brand	2018	What brand of e-cigarette/vaping device…	Prompted to start typing the name of the brand to see a list of relevant brand names and find a match. If brand not listed, please select 'Other' and type the name of your brand.
NVP Brand	2020	What brand of vaping device…	Same as 2018 for disposable/cartridge users.<br>Tank users were asked brand open-ended, without providing a brand list.
Adjustable Voltage	2018,	Can you adjusted the power, voltage, or temperature…	1) Yes, but I don't change it<br>2) Yes, and I change the settings occasionally

<table>
<tr><td></td><td></td><td>Asked to cartridge/tank only</td><td colspan="2">3) Yes, and I regularly adjust the settings
4) No
5) Refused
6) DK</td></tr>
<tr><td>Adjustable Voltage</td><td>2020</td><td>Can you adjust the power and voltage…</td><td colspan="2">1) Yes
2) No
3) Refused
4) DK</td></tr>
<tr><td>NVP capacity</td><td>2018, 2020</td><td>What is the volume/capacity of the tank/cartridge…

Asked to cartridge/tank only</td><td colspan="2">1) Less than 1 ml
2) 1 – 1.5 ml
3) 1.6 – 2.0 ml
4) 2.1 – 3.0 ml
5) 3.1 – 4.0 ml
6) Over 4 ml
7) Refused
8) DK</td></tr>
<tr><td>Nicotine Content</td><td>2018</td><td>What is the nicotine strength of the e-liquid</td><td colspan="2">1) No nicotine - 0 mg/ml (0%)
2) 1 – 4 mg/ml (0.1 – 0.4%)
3) 5 – 8 mg/ml (0.5 – 0.8%)
4) 9 – 14 mg/ml (0.9 – 1.4%)
5) 15 – 20 mg/ml (1.5 – 2.0%)
6) 21 – 24 mg/ml (2.1 – 2.4%)
7) 25 mg/ml (2.5%) or more
8) Refused
9) DK</td></tr>
<tr><td>Nicotine Content</td><td>2020</td><td>1) Choose how you want to report nicotine strength, by % of mg/ml
2) What is the nicotine strength….</td><td>1) None (0%)
2) Less than 1%
3) 1 to 1.9%
4) 2 to 2.9%
5) 3 to 3.9%
6) 4 to 4.9%
7) 5% or more
8) Refused
9) DK</td><td>1) None (0 mg/ml nicotine)
2) Less than 10 mg/ml
3) 20 – 29 mg/ml
4) 30 – 39 mg/ml
5) 40 – 49 mg/ml
6) 50 mg/ml or more
7) Refused
8) DK</td></tr>
<tr><td>Nicotine Salt</td><td>2020</td><td>Have you ever used the salt form of nicotine e-liquid?</td><td colspan="2">1) Yes
2) No
3) Refused</td></tr>
</table>

			4) DK
NVP Consumption	2018, 2020	(Disposable) On average, about how many disposable vaping devices do you now use each week?<br><br>(Cartridge) On average, about how many cartridges or pods do you now use each week?<br><br>(Tank) How many days will this amount last – after being assed about last e-liquid bottle purchase	Open-ended question with Refused and DK options<br><br>1) Less than 1 day<br>2) Open: type number of days<br>3) Refused<br>4) DK

Note: 2018 did not include the term “pods” while 2020 referred to “cartridges or pods.”

Supplemental Table S2. DK percentages by country.

	Canada	*United States*	*England*	*Australia*		
n at 2018	*901*	*992*	*2037*	*263*		
n at 2020	*1208*	*879*	*1555*	*252*	X^2 *or F*	*p*
NVP Description						
2018	0.2	0.7	0.1	0.8	7.96	0.045
2020	0.1	0.0	0.2	0.0	2.45	0.49
NVP Appearance						
2018	0.0	0.0	0.1	0.0	2.12	0.55
2020	0.2	0.3	0.3	0.0	0.96	0.81
Brand						
2018	23.4	12.8	10.9	6.6	93.15	< .001
2020	6.9	11.1	9.5	10.9	12.14	0.007
Adjustable Voltage						
2018	3.5	1.1	2.1	2.4	11.65	0.009
2020	6.3	2.8	4.4	4.4	13.21	0.004
Capacity						
2018	27.6	34.3	31.4	11.6	52.73	<.001
2020	26.1	31.6	26.4	14.9	27.24	<.001
Nicotine Content						
2018	15.4	9.8	6.7	5.7	61.29	< .001
2020	5.2	9.7	6.7	13.6	30.54	< .001
Nicotine Salt						
2018	n/a	n/a	n/a	n/a	n/a	n/a
2020	4.6	4.4	7.4	4.0	15.52	0.001
Consumption						
2018	10.0	9.2	10.2	6.4	4.17	0.37
2020	4.0	8.5	6.6	17.2	28.49	<.001
DK Index						
2018	11.39 (0.49)	9.31 (0.39)	8.69 (0.26)	5.03 (0.65)	17.46	<.001
2020	6.71 (0.29)	8.23 (0.37)	7.75 (0.29)	7.29 (0.79)	5.32	0.001
